# The importance of corneal assessment in a glaucoma suspect – a review

**Published:** 2019

**Authors:** Vasile Potop, Cătălina Corbu, Valeria Coviltir, Speranţa Schmitzer, Mihaela Constantin, Miruna Burcel, Cătălina Ionescu, Dana Dăscălescu

**Affiliations:** * “Carol Davila” University of Medicine and Pharmacy Bucharest, Romania; ** Clinical Hospital of Ophthalmologic Emergencies Bucharest, Romania; *** Oftaclinic Bucharest, Romania

**Keywords:** glaucoma, corneal properties, intraocular pressure

## Abstract

Glaucoma represents the main cause of irreversible blindness in the world and for this consideration, the interest in a quick and precise diagnosis and progression of the disease, prior to the appearance of irreversible damage, has been continuously rising.

Glaucoma risk factors are already well known, but current studies reveal that it is necessary to make a proper analysis of the intraocular pressure (IOP) to obtain an accurate diagnosis, so we must take into consideration corneal properties that might affect IOP measurements.

Starting from corneal geometrical properties represented by central corneal thickness (CCT) and continuing with biomechanical properties represented by corneal hysteresis (CH) and corneal resistance factor (CRF) we reviewed the value of investigating corneal properties in ocular hypertension (OH), primary open angle glaucoma (POAG) and normal tension glaucoma (NTG) patients. We can now say that CCT plays an important role in diagnosing glaucoma because it may mask the real value of the IOP and also, in setting the target for the IOP needed to stop disease progression. Also, CH is a factor that needs to be screened from the first consult of a glaucoma patient or suspect because it is correlated to the response to treatment, visual field (VF) and retinal nerve fiber layer (RNFL) progression and could anticipate the future evolution and patients prognosis.

Both CCT and CH are factors that must be thought-about when we encounter a glaucoma suspect. CCT has a predictive role in OH and NTG patients, while CH has on the other hand a prognostic role in POAG, OH and NTG patients.

## Introduction

Glaucoma represents a disease with an important social impact due to the chronic treatment that has high costs and irreversible RNFL changes with consequences on the visual field. It is critical to determine which patients have a high risk of progression if we want to preserve their visual acuity (VA) and to prevent VF loss.

Initially, OH and POAG patients have no symptoms and the ophthalmologist suspects the diagnosis during a routine examination. This makes glaucoma one of the most complicated and complex diseases, especially regarding the progression control, because the patients deny their diagnosis, ignore the risks, and do have compliance to treatment until symptoms appear. At that point, we can only prevent the display of new VF changes.

Moreover, considering the fact that there are no clinical symptoms, patients sometimes present late, in advanced stages, with decreased VA and irreversible VF loss. Knowing that an established VF defect cannot be reversed, we have to diagnose the disease early and to recognize those patients who have a high risk of progression. 

## Corneal properties

Cornea can be defined by a sum of physical, geometrical (shape, topography and thickness) and biomechanical properties (CH and CRF) [**1**]. 

The interest in corneal biomechanics was initially regarding its response in refractive surgery, but later, more and more studies revealed their involvement in keratoconus and glaucoma [**1**-**5**].

Many studies showed that local factors could influence IOP measurements: CCT, corneal rigidity and shape or axial length [**6**-**13**].

Regarding corneal properties, physical properties have been investigated more than biomechanical properties, probably because of the lack of instruments that could measure it in vivo [**1**]. Initially, hypotheses stated that corneal properties might have a role in the disease pathogenesis. This fact was then confirmed by clinical studies that proved the impact of CCT variability on the optic nerve head. This implied that corneal properties could have an independent role in the progression of glaucoma [**1**,**13**,**14**].

Today, devices are capable of measuring not just CCT, but also corneal viscoelastic properties, so the interest in corneal biomechanical properties has been rising and the importance of the corneal hysteresis in glaucoma was revealed [**15**]. These devices are also capable to adjust IOP measurements based on CH and CRF and to provide a corneal compensated IOP measurement not as much influenced by corneal properties, making it more accurate [**1**,**15**-**17**]. 

## Central corneal thickness and glaucoma

In the past, many patients have been misclassified as glaucomatous or non-glaucomatous based on their measured IOP. Time proved that this classification was insufficient because many non glaucomatous patients developed VF defects, while some glaucomatous patients remained stable a long time [**18**,**19**]. Today, we know that CCT measurement is valuable due to the fact that it can mask an accurate reading of eye pressure [**18**,**19**]. Values lower than 555 microns may show artificially low IOP.

Thinner CCT can be encountered more frequently in patients with more advanced glaucoma or NTG and is considered an independent risk factor in OH [**18**,**19**]. The predictive value of the CCT in case of patients with established glaucoma has not been demonstrated. Nonetheless, it is important in clarifying the IOP results, in risk stratification and establishing target IOP [**18**,**19**].

Studies revealed that CCT was lower in the eyes of glaucoma patients than in normal eyes. The Ocular Hypertension Treatment Study (OHTS) determined that a thin CCT is a very important factor that can predict the transformation of OH in POAG [**18**,**20**]. In OHTS, a 5 year follow-up showed that patients with CCT lower than 555 microns had 3 times the risk of developing the disease in comparison with a subject with a CCT higher than 588 µm [**18**,**20**]. 

In patients diagnosed with preperimetric glaucoma, CCT represents an important risk factor for the development of VF loss. It is vital to take into consideration CCT when we establish the target IOP for each patient with glaucoma [**18**,**21**,**22**]. 

Based on the results from the OHTS and the European Glaucoma Prevention Study, experts developed a method that estimates the individual 5-year risk of a patient with OH to develop POAG [**18**,**20**,**23**]. 

## Genetic involvement in Central Corneal Thickness 

Nowadays, studies revealed a genetic relationship between corneal thickness and glaucoma. It appears that the thickness of the cornea is genetically predetermined [**24**]. 

Human studies like Genome-wide association studies identified loci that are associated with CCT on chromosomes 1 (near genes COL8A2), 9 (near genes ZNF469), and 16 (near genes RXRA/ COL5A1). More than 11 single nucleotide polymorphisms (SNPs) have been linked to a decreased CCT [**24**]. 

A study conducted on mice found that variations in the genes which code for a protein POU6F2 correlated with CCT [**24**,**25**]. POU6F2 is found in retinal ganglion cells and in corneal limbal stem cells. The distribution of protein POU6F2 within the developing eye reveals a connection between the cornea and the retinal ganglion cells during their development [**25**]. Studies showed that this protein is associated to a high susceptibility to injury of retinal ganglion cells [**25**]. POU6F2 is involved in the development of the cornea [**25**]. When the researchers modified the gene that codes for POU6F2, the corneas of the mice with modified genes were thin, whereas normal mice had normal CCT [**25**].

## Corneal biomechanical properties and glaucoma

Corneal biomechanical properties can be measured ex vivo using destructive techniques or in vivo using either Ocular Response Analyzer (ORA) (Reichert, Buffalo, New York, USA) or Corneal Visualization Scheimpflug Technology ST (Corvis ST) (Oculus Optikgerate GmbH, Wetzlar, Germany [**26**-**31**].

ORA is a device that generates corneal biomechanical parameters measured in vivo [**26**. This test is noninvasive, quick, cheap, and easy to perform [**26**]. It could help us not only to diagnose glaucoma, but also to identify patients with a higher risk of progression even from the first consult [**32**]. ORA generates four parameters: IOPcc (corneal compensated intraocular pressure), IOPg (Goldmann related intraocular pressure), CH (corneal hysteresis) and CRF (corneal resistance factor) [**26**,**33**]. IOPcc is a new IOP measurement compensated and uninfluenced by corneal properties [**1**,**33**-**35**]. IOPg is an IOP measurement comparable to Goldmann measured IOP [**1**,**33**-**35**]. 

CH probably represents the most important parameter generated by ORA. It refers to a dynamic behavior of the cornea measuring its viscoelastic properties. It is a parameter to consider in glaucoma patients [**1**,**2**,**33**-**36**]. 

CRF is an symbol of the whole corneal resistance and is probably more useful in corneal pathology such as keratoconus or pellucid marginal degeneration [**1**,**33**-**35**].

There is a direct connection between corneal physical and biomechanical properties, especially between CRF and CCT where a positive correlation was proved [**37**,**38**].

It was demonstrated that a raised IOP is correlated to a low CH and the other way around. Moreover, at the same value of the IOP, CH is has a lower value in POAG patients than in OH patients and is even lower than in NE [**39**].

Probably the most crucial role of corneal biomechanical properties is played in OH patients [**15**,**20**,**36**,**38**]. Both CH and CRF are underexpressed in glaucoma patients comparing to OH patients and in these patients more than in NE. Also, in patients with asymmetric disease, in the most affected eye we encounter a lower CH compared to the other eye and eyes that have a high CH react better in front of IOP variations [**15**,**20**,**36**,**38**].

Considering that an eye with decreased viscoelastic behavior might have a more vulnerable optic disc to raised IOP, studies revealed that CH, but not CCT, is connected to an increased deformation of the surface of the optic nerve head during transient IOP elevations[**4**,**31**,**40**]. The direct relationship between CH and RNFL and VF parameters show us that is important to measure CH as well as we measure many other parameters in every glaucoma suspect [**4**,**31**]. 

Authors revealed that CH might be a parameter linked to disease progression. A low CH in a patient with glaucoma represents a risk factor for VF loss in a 5-year period. A decrease in the CH value is followed by a drop in VFI and if that glaucoma patient associates a high IOP, the risk increases [**22**,**41**-**43**].

## Conclusions

In front of a patient suspect of glaucoma, we should consider that one examination cannot be sufficient to confirm this diagnosis, so, along with IOP, VF, OCT and HRT we could also use corneal properties. 

CCT measurement may be a vital exam when assessing a patient who is suspect of glaucoma. Also, the appropriate target IOP in patients with NTG or POAG, could be different than what we might consider safe.

CH may characterize corneal properties more thoroughly than CCT alone. It may also be a parameter that better predicts the progression of the disease. 

In a glaucoma suspect, combining CH and CCT for the evaluation of the glaucoma risk improves diagnostic capability compared to using either factor alone. Measuring CH and CCT in the first consult of a subject with glaucoma or suspect could help us identify patients who have a higher risk of disease progression.

Also, these parameters can help evaluate if a patient who appears to be stable needs a closer monitorization, but we should not base our therapeutic decision on corneal properties alone.

More extensive studies have to be performed in order to demonstrate the exact relevance of corneal properties in glaucoma patients.

**Acknowledgment**

All authors have equally participated to this paper, having the same contribution, with the first author. 
